# Smart Implantable Device-Enabled Remote Monitoring Reduces Rates of Manipulation Under Anesthesia Following Total Knee Arthroplasty

**DOI:** 10.7759/cureus.95376

**Published:** 2025-10-25

**Authors:** John Dundon, Nicholas Brown, Monika Wlodarski, Brian Begley

**Affiliations:** 1 Orthopedic Surgery, Tri-County Orthopedics, Cedar Knolls, USA; 2 Orthopedics, Orthopedic Research Institute of New Jersey, Bridgewater, USA; 3 Orthopedics, Tri-County Orthopedics, Cedar Knolls, USA; 4 Orthopedics, Atlantic Health System, Morristown, USA

**Keywords:** manipulation under anesthesia, orthopedic anesthesia, orthopedic surgery, smart implantable device, total knee arthroplasty

## Abstract

Background

Total knee arthroplasty (TKA) is a highly successful procedure; however, postoperative joint stiffness remains a common complication, occasionally requiring manipulation under anesthesia (MUA). This retrospective study aimed to compare MUA incidence between patients who received smart implantable devices (SIDs) and those with traditional components following TKA. We hypothesized that the use of SIDs through remote monitoring would result in decreased MUA incidence when compared to traditional knee implants.

Methods

This study included 806 patients who underwent primary TKA by a high-volume surgeon between July 2023 and December 2024. Of these patients, 500 received traditional implants, while 306 received SIDs. A power analysis was performed to determine the required sample size. The analysis indicated that a minimum of 431 patients would be required per group. Patients were followed for at least 90 days postoperatively, and MUA incidence was recorded. The demographic data and the Knee Injury and Osteoarthritis Outcome Score, Joint Replacement (KOOS, JR) measures were analyzed.

Results

The incidence of MUA was 3.101% for traditional implant recipients and 0.971% for SID recipients (p<0.05). Traditional implant recipients were over three times more likely to require MUA.

Conclusion

The use of SIDs in this study demonstrated a significant reduction in MUA incidence following TKA. SID implantation with remote monitoring led to a threefold decrease in the rate of MUA. This study highlights the potential of SIDs to significantly reduce the rate of MUA through remote monitoring of several gait metrics, such as functional range of motion, cadence, and step count, supporting their integration into TKA care protocols.

## Introduction

Total knee arthroplasty (TKA) is a highly successful and highly utilized procedure in the United States. The annual volume of TKA has increased by 156% from 2000 to 2019 and is expected to surpass 1.2 million cases per year by 2040 [1]. Despite the high success rates, patient dissatisfaction remains high, with only 80-90% of patients pleased with their outcomes. Approximately 50% of patients report at least one complication following TKA [2]. Joint stiffness is one of the most common complications following TKA and can have a severe impact on patient recovery [3]. The prevalence of postoperative stiffness slightly varies in the literature, having been found to range from 2.6 to 4% within the last decade [4-6]. The definition of stiffness continues to evolve and is not universally accepted. Recent literature has defined postoperative stiffness as lacking 10 degrees of terminal extension, less than 90 degrees of maximum flexion, or a decreased range of motion (ROM) compared to preoperatively [6-9]. 

Postoperative stiffness is commonly caused by arthrofibrosis, in which patients experience limited ROM caused by excessive scar tissue [10]. Options of treatment for postoperative stiffness include physical therapy, manipulation under anesthesia (MUA), debridement (both arthroscopic and open), and revision surgery. When progress with ROM following physical therapy halts, MUA is typically the primary treatment method for stiffness following TKA [11]. Patients who have undergone previous ligamentous surgeries, have a limited preoperative ROM, are young, are female, and have poor postoperative rehabilitation are most at risk for developing arthrofibrosis [12,13]. Although there seems to be no clear consensus on the optimal timing for intervention, some studies have detailed improved outcomes following MUA when performed earlier [11].

The timing of intervention is often limited by the frequency of surgeon-patient interaction, limited measurement capability, and a lack of defined algorithms for when to proceed with MUA. Furthermore, the increased demand for TKA and decreased reimbursement have created increasing time pressures, necessitating the need for expeditious, accurate, and detailed data measures. Patient-reported outcome measures (PROMs), like the Knee Injury and Osteoarthritis Outcome Score, Joint Replacement (KOOS, JR), have been relied on to provide objective insight in evaluating the success of a patient following TKA; however, these measures are subjective, and there is variability depending on individual patient reporting [14].

In recent years, remote monitoring through wearable devices and smart implantable devices (SIDs) has allowed for more objective data analysis, such as step count, cadence, and stride length, in postoperative care and has been noted for its ability to provide a wide range of accurate data [15]. Research documenting the clinical utility of this data is still lacking, and the impact on patient care has not yet been documented. The cost of a TKA varies depending on the implant design, technology used, and type of medical insurance. A conventional TKA implant typically has a lower cost for the implant and instrumentation used when compared to SIDs. The SID implant and instrumentation may be more costly up front but can reduce episode-of-care costs post-procedure [16]. The goal of this study was to compare the MUA incidence in patients who received either SID or traditional components following TKA. We hypothesized that there would be a decrease in MUA for patients implanted with SIDs.

## Materials and methods

Ethics

This study included patients who underwent primary TKA by a single high-volume attending surgeon, as well as any patients who underwent subsequent MUA following TKA completed by this surgeon. IRB approval was obtained prior to data collection for this study through WCG Clinical, Inc., with an exemption for HIPAA authorization number 1-182327-1 on November 26, 2024.

Study design

To minimize concerns about patient selection bias and manipulation rates, MUA rates for the surgeon implanting SIDs were included. The differences in MUA rates were assessed. The SIDs did not interact with the joint space or surrounding soft tissue in any altered way when compared with the traditional prosthesis. The SID placed was a Persona IQ Zimmer Biomet device. The SID automatically collects gait metrics, including ROM, step count, walking speed, and stride length, and other data throughout the day. The implant stores data for up to 30 days via wireless transmission, which is uploaded to a secure, HIPAA-compliant cloud-based platform each night. The data are uploaded to the mymobility app. Alerts are sent within the system to support remote patient monitoring (RPM), which can be viewed by the patient and care team. These alerts include app notifications and allow the provider to see if the patient is falling below a 25% threshold. The surgeon used robotic arm assistance during surgery with a restrictive kinematic alignment technique. Only MUAs following primary TKA were included. Patients were required to have at least a 90-day follow-up to be included in the study to allow for proper postoperative monitoring. Routine follow-ups were obtained at 2-week, 6- to 8-week, and 3-month intervals. Those who did not complete at least a 3-month follow-up were excluded from the study.

Patient population

**Figure 1:**
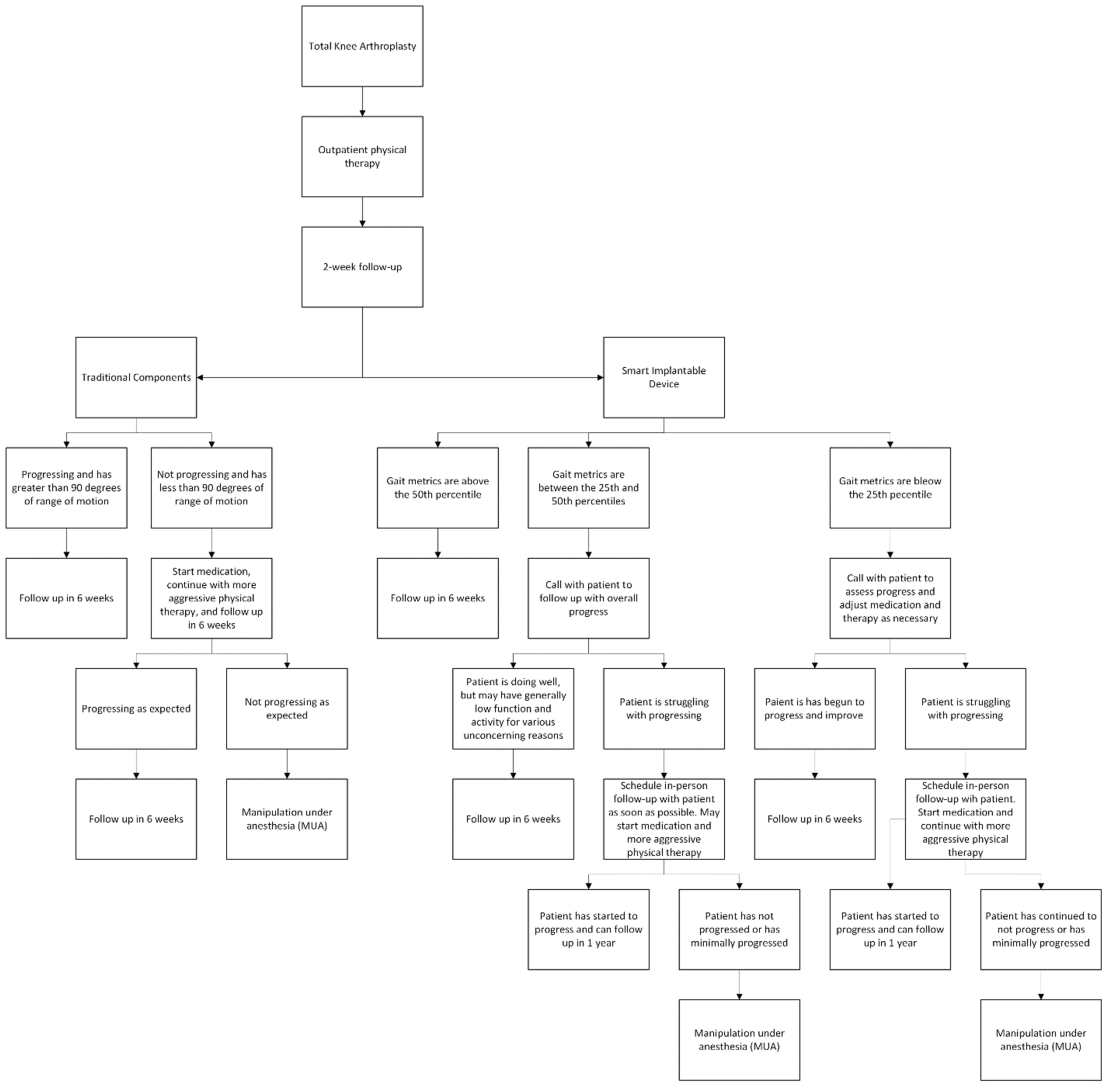
Flowchart describing the protocol and timeline for core following primary total knee arthroplasty.

Between July 10, 2023, and December 31, 2024, 500 primary TKAs were identified without SID. There were 306 patients in the SID group with at least 90 days of follow-up recorded, giving a total of 806 cases for evaluation. Patients were indicated for MUA if they presented with stiffness or a limited ROM (less than 90 degrees ROM after 6 weeks and up to 3 months) (Figure 1).

Enrollment criteria

All patients in both groups had access to a patient care management (PCM) platform with access to daily text reminders, a home exercise program, patient information sessions, and direct text messaging capability with the care team. The scheduled and routine follow-up was the same for both cohorts. In the SID group, along with the routine follow-up, their findings and what their gait data implied with their recovery, compared to their own national cohort, were discussed. Daily monitoring of the data was performed by one of the members of the care team (MD, RN, NP, or PA), and these members monitor the SID results. Alterations in gait parameters led to intervention by one of the team members, who incited either a call, text, or in-person visit. Typically, multiple parameters below the 20th percentile for their age and sex cohort for more than three consecutive days prompted intervention by the care team.

Data collection

This study examined the MUA incidence rate for patients who received SIDs and non-SIDs. KOOS, JR scores were also of interest in order to determine patient stiffness, pain, and knee function. The scale for KOOS, JR is 0-28 and is adjusted to 0-100. KOOS, JR is a PROM, where higher adjusted scores or lower raw scores relate to increased satisfaction and knee function. KOOS, JR scores were obtained preoperatively and 90 days postoperatively. Demographics were also recorded to ensure no difference between patient cohorts. The significance level for this study was set at p<0.05.

Fisher's exact tests were used to identify any significant differences in MUA incidence rates between TKAs, where patients received SIDs or non-SIDs. Fisher's exact tests (one-sided) were also used to determine any relationship in categorical data between the two groups, such as sex and race. Statistical significance was set at p<0.05.

Statistical analysis

No significant differences in sex, race, and body mass index (BMI, kg/m²) were observed between the MUA and non-MUA groups for both implant types. Those who underwent an MUA in either group were, on average, significantly younger than those who received the same implant type.

A power analysis was performed to determine the required minimum sample size to detect a statistically significant difference in MUA between SID and conventional implants. Using an alpha level of 0.05 and a desired power (1-beta) of 0.80, the analysis indicated that a minimum of 431 patients would be necessary for each group.

## Results

A total of 806 patients were identified from the practice's electronic health record; 500 (62.0%) received standard implants, whereas 306 (38.0%) received SIDs. Those who received traditional implants underwent MUA (16 patients), leading to a 3.101% incidence rate. Of those who received SIDs, three patients underwent MUA, with a 0.971% incidence rate for this sample. The Fisher's exact test revealed a significant decrease in MUA incidence for the SID group (p<0.05) (Table 1).

**Table 1:** Comparison of manipulation under anesthesia (MUA) rates for the smart implantable device (SID) cohort and standard, traditional cohort. Fisher's exact test was used with significance set to p<0.05.

Implant Cohort	MUA (n)	No MUA (n)	Incidence (%)	P-value
SID	3	306	0.971	0.048
Traditional	16	500	3.101	

Mixed results were achieved when analyzing PROMs for both groups. No statistical difference was found for preoperative KOOS, JR scores between those who had an MUA and those who did not, regardless of the implant type (p>0.05) (Table 2). Those who received traditional implants but did not undergo an MUA exhibited significantly higher 12-week postoperative KOOS, JR scores than those who underwent an MUA with the same implant type (p<0.005). This result was not true for the SID cohorts.

**Table 2:** Demographics and patient-reported outcome measures. Fisher's exact tests were used to compare categorical variables, and Student's t-tests were used to compare continuous variables. Significance was set to p<0.05. SID: smart implantable device; MUA: manipulation under anesthesia; SD: standard deviation; KOOS JR: Knee Injury and Osteoarthritis Outcome Score, Joint Replacement

Demographics	SID	SID	P-value	Traditional	Traditional	P-value
	MUA (n=3)	No MUA (n=303)		MUA (n=16)	No MUA (n=500)	
Age, mean±SD (years)	55.7±13.1	66.0±8.17	0.031	59.6±13.5	69.9±8.56	<0.0001
Sex, n (%)						
Female	1 (33.3)	169 (55.2)	0.59	11 (64.7)	314 (62.8)	1
Male	2 (66.7)	137 (44.8)		6 (35.3)	186 (37.2)	
Race, n (%)						
White	3 (100)	252 (82.4)	1	15 (88.2)	437 (87.4)	0.658
Black	0 (0)	4 (1.31)		0 (0)	11 (2.2)	
Asian	0 (0)	4 (1.31)		1 (5.9)	15 (3.0)	
Other	0 (0)	46 (15.4)		1 (5.9)	37 (7.4)	
Body Mass Index, mean±SD (kg/m²)	30.9±6.86	30.5±5.66	0.905	29.9±5.1	29.9±5.54	1
KOOS JR, interval, mean±SD						
Preoperative	46.0±14.1	49.5±13.9	0.665	45.2±12.7	51.2±13.2	0.066
12-Week Postoperative	77.7±9.83	67.1±11.6	0.118	60.6±9.0	68.8±11.6	0.005

## Discussion

This retrospective cohort study found a significant decrease in the MUA rate with SID-implanted knee replacements compared with traditional knee replacements. A three- to fourfold decrease in MUA rates in our TKA population was observed following the implementation of a SID and remote monitoring. MUA incidence rates across all groups in this study are comparable to published averages, suggesting that component usage and surgeon performance had minimal impact on the study's outcomes [6]. No significant difference in MUA rates with traditional implants was observed. This study suggests that SID implementation and remote monitoring, along with early patient intervention, may help reduce the rate of MUA and complications in our TKA population.

While MUA may be considered a minor procedure, it has a significant impact on costs during the 90-day episode of care and on the risk of future revision [17]. MUA also led to an increased incidence of revision surgery and PJI [6]. Patients undergoing MUA were 2.4 to 2.9 times more likely to require revision surgery [18,19]. Revision TKA is associated with a significant increase in patient and health care costs and morbidity, with costs increasing as additional components are revised [20]. This cost is further exaggerated by extended hospital lengths of stay that revision TKA patients typically endure. Although MUA performed within three months of TKA has been found to increase ROM and functional scores, its impact on the healthcare system and cost to the patient should be considered [21].

We believe that the decrease in MUA incidence for those who receive SIDs is mainly due to the ability to remotely monitor patient recovery through the various metrics provided by these devices. The device used in our practice allows for data tracking of various outcome measures, such as qualified step count, distance traveled, functional ROM, stride length, and cadence. By monitoring these metrics, we are able to alter our approach to recovery to best suit the patient. This has led to early intervention in several patients, potentially decreasing the risk of stiffness and arthrofibrosis, and significantly reducing the rate of MUA. Interventions included alteration of PT, anti-inflammatories, reassurance, and improved pain control.

With the increased utilization and integration of artificial intelligence (AI) in the medical field, particularly in orthopedics, much of the intervention process may be automated in the future. Declining reimbursement and increased administrative burden, combined with increased personnel costs, are forcing increased efficiency in orthopedic practices. To survive in this environment, practices, including medical assistants, surgeons, and nursing assistants, will be compelled to utilize technology to redirect care and resources to patients who need them most. Without high-quality source data, this transformation will be difficult to navigate.

This study is not without its limitations. This study is underpowered due to a low incidence of manipulation; increasing the number of patients would certainly improve the statistical significance. Given the low incidence of manipulation in this study, the statistical fragility could be significantly changed by one or two manipulations in either group. This is a new technology, and there is no clear-cut defined pattern to delineate concern for these patients; hence, this can be highly user-dependent data. Because of this, data analysis is currently highly user-dependent, and this study may not be applicable to all surgeons; therefore, further research is needed to better define and determine the gait patterns.

In this study, there is significant concern about selection bias due to its retrospective design, and it is impossible to blind either the patients or the surgeons. Only one surgeon has used the SID technology, raising concerns that it may not be applicable to all surgeons.

## Conclusions

In conclusion, a potentially significant decrease in the MUA rate between SID implants with remote monitoring and traditional TKA implants was observed, supporting our hypothesis. The incidence of MUA among patients with SID was 0.971%, while traditional implant patients had a 3.101% incidence rate. Unfortunately, patients who underwent MUA were also 2.4 to 2.9 times more likely to require a revision TKA, which increases long-term costs for these patients. Future studies are warranted to determine the general applicability of this technology and confirm these findings. Additionally, markers concerning the risk of MUA should be monitored and measured.
